# Molecular Docking and Dynamics Studies to Explore Effective Inhibitory Peptides Against the Spike Receptor Binding Domain of SARS-CoV-2

**DOI:** 10.3389/fmolb.2021.791642

**Published:** 2022-01-27

**Authors:** Suvro Biswas, Shafi Mahmud, Mohasana Akter Mita, Shamima Afrose, Md. Robiul Hasan, Mst. Sharmin Sultana Shimu, Md. Abu Saleh, Gomaa Mostafa-Hedeab, Mohammed Alqarni, Ahmad J. Obaidullah, Gaber El-Saber Batiha

**Affiliations:** ^1^ Department of Genetic Engineering and Biotechnology, University of Rajshahi, Rajshahi, Bangladesh; ^2^ Pharmacology Department and Health Research Unit-medical College, Jouf University, Jouf, Saudi Arabia; ^3^ Pharmacology Department, Faculty of Medicine, Beni-Suef University, Beni Suef, Egypt; ^4^ Department of Pharmaceutical Chemistry, College of Pharmacy, Taif University, Taif, Saudi Arabia; ^5^ Drug Exploration and Development Chair (DEDC), Department of Pharmaceutical Chemistry, College of Pharmacy, King Saud University, Riyadh, Saudi Arabia; ^6^ Department of Pharmacology and Therapeutics, Faculty of Veterinary Medicine, Damanhour University, Damanhour, Egypt

**Keywords:** SARS-CoV-2, peptides, RBD, peptide-protein docking, molecular dynamics

## Abstract

The spread of severe acute respiratory syndrome coronavirus 2 (SARS-CoV-2) has become a pandemic due to the high transmission and mortality rate of this virus. The world health and economic sectors have been severely affected by this deadly virus, exacerbated by the lack of sufficient efficient vaccines. The design of effective drug candidates and their rapid development is necessary to combat this virus. In this study, we selected 23 antimicrobial peptides from the literature and predicted their structure using PEP-FOLD 3.5. In addition, we docked them to the SARS-CoV-2 spike protein receptor-binding domain (RBD) to study their capability to inhibit the RBD, which plays a significant role in virus binding, fusion and entry into the host cell. We used several docking programs including HDOCK, HPEPDOCK, ClusPro, and HawkDock to calculate the binding energy of the protein-peptide complexes. We identified four peptides with high binding free energy and docking scores. The docking results were further verified by molecular dynamics (MD) simulations to characterize the protein-peptide complexes in terms of their root-mean-square fluctuation (RMSF), root-mean-square deviation (RMSD), radius of gyration (Rg), solvent-accessible surface area (SASA), and hydrogen bond formation. Allergenicity and toxicity predictions suggested that the peptides we identified were non-allergenic and non-toxic. This study suggests that these four antimicrobial peptides could inhibit the RBD of SARS-CoV-2. Future *in vitro* and *in vivo* studies are necessary to confirm this.

## Introduction

The whole world is currently experiencing a pandemic which originated in the Chinese city of Wuhan in Hubei province in late December 2019. This life-threatening agent was named severe acute respiratory syndrome coronavirus 2 (SARS-CoV-2) by WHO, which declared it as “the first pandemic of the 21st century” (De Wit et al., 2016; Dong et al., 2020; Gorbalenya et al., 2020; Li Q et al., 2020; Li X et al., 2020; Machhi et al., 2020; Zhu et al., 2020). SARS-CoV-2 is linear single-stranded positive sense enveloped RNA virus which contains a crown-like spike on its surface. SARS-CoV-2 has a genome size ranging from 26 to 32 kilobases and a virion size of roughly 80–120 nm in diameter (Li et al., 2005; Cui et al., 2019; Chen, 2020; Gorbalenya et al., 2020; Lu et al., 2020; Machhi et al., 2020; Wrapp et al., 2020; Yin, 2020). At present, 224 countries and territories are affected by SARS-CoV-2 viral infection. As of November 16, 2021, there have been a total of 254,807,373 confirmed cases and 5,126,239 deaths (https://www.worldometers.info/coronavirus/). SARS-CoV-2 is considered the third most highly pathogenic coronavirus. Its genome encodes four structural proteins: a helical nucleocapsid protein (N), an envelope protein (E), membrane/matrix protein (M) which has a significant role in viral assembly, and the spike surface glycoprotein (S), which facilitates viral entry into the host cell (Ashour et al., 2020; Dehelean et al., 2020; Khan et al., 2020). Several studies suggest that SARS-CoV-2 is zoonotic in origin, with 79.9% nucleotide sequence identity with SARS-CoV, 51.8% identity with MERS-CoV, and 87.6–89% identity with the bat-origin SARS-like coronavirus (bat-SL-CoVZC45) (Dehelean et al., 2020; Machhi et al., 2020; Ren et al., 2020; Wu et al., 2020; Zhang and Holmes, 2020).

The S proteins of coronavirus consist of spike monomers with two subunits, S1 and S2 (Gui et al., 2017; Yuan et al., 2017; Kirchdoerfer et al., 2018; Song et al., 2018; Lan et al., 2020). The S1 subunit contains the receptor-binding domain (RBD) and N-terminal domain (NTD) which are responsible for virus binding and entry. The RBD is located in the middle part of the S1 subunit and is used as an antigen to raise antibodies that interrupt virus-host binding (Xiao et al., 2003; Babcock et al., 2004; He et al., 2004; Wong et al., 2004; Lan et al., 2020; Dejnirattisai et al., 2021). The S2 domain has a proposed fusion peptide and two heptad repeats (HR1 and HR2) that facilitate cell membrane fusion between viral and target cells following proteolytic activation (Wild et al., 1994; He et al., 2004; Shang et al., 2020; Kim et al., 2021). The RBD-containing S1 domain also contains the SD1 and SD2 subdomains at the C-terminus. Although both the NTD and the RBD are immunogenic, the RBD is contains the interaction surface for ACE2. Due to the fact that the receptor-binding site (RBS) is incompletely driven into the down state, the RBD solely engages with the up state of ACE2 (Lan et al., 2020; Premkumar et al., 2020; Dejnirattisai et al., 2021; Yuan et al., 2021).

Usually, many years of research are required before vaccines enter clinical trial. However, in a record period, scientists and researchers have made great efforts to develop secure, efficient, and active SARS-CoV-2 vaccines. Currently, 13 vaccines have been approved for early or limited use, and 8 vaccines have been approved for complete use (https://www.nytimes.com/interactive/2020/science/coronavirus-vaccine-tracker). On December 31, 2020, the WHO prepared an emergency use listing (EUL) for a vaccine named ‘BNT162b2/COMIRNATY Tozinameran (INN)’ manufactured by Pfizer. SK Bio and the State Institute of India generated “AZD1222” and “Covishield” vaccines that received an EUL on February 16, 2021. The “Ad26. COV 2. S” developed by Janssen (a subsidiary of Johnson & Johnson) was displayed on March 12, 2021. Moderna developed the “mRNA-1273” vaccine. The Sinopharm vaccine and the Sinovac-CoronaVac have also been granted EUL by the WHO [https:// www.who.int/news-room/q-a-detail/coronavirus-disease-(covid-19)-vaccines]. To date, only 2.2% of people in low-income countries have had at least one SARS-CoV-2 vaccine dose (https://ourworldindata.org/covid-vaccinations).

In this study, the selected peptides were docked to the RBD of SARS-CoV-2, leading to the identification of four peptides with high binding free energy. These peptide-RBD complexes were subsequently subjected to molecular dynamics study. Structural attributes and conformations of the docked complexes were obtained from the MD simulations, and suggested stiff and inflexible interactions between the RBD active site and the hit peptides. In comparison to earlier studies, we utilized multiple docking programs in combination to identify four peptides with high binding affinity to the active site of the RBD. Several previous studies have suggested antiviral effects of small molecules and peptides against SARS-CoV-2 through binding to the RBD. These studies identified molecules that, although they were predicted to bind to the RBD, did not interact directly with the RBD active site (Rathod et al., 2020; Padhi et al., 2021; Priya et al., 2021). In contrast, all of the peptides we identified were predicted to bind directly to the RBD active site. Although a few previous studies identified peptides that formed a single non-bonded interaction with the RBD active site, the predicted binding energies were lower than ours using the same docking software (Chowdhury et al., 2020; Hossain et al., 2021) and the complexes were less stable in MD simulations.

## Materials and Methods

### Peptide Screening and Preparation

In this study, we started from 27 peptide molecules that were previously identified in the venom of the wild bee *Hylaeus signatus* and which were screened for antimicrobial activity (Nešuta et al., 2016). Three peptides were excluded as they contained D-amino acids. Additionally, one further peptide was excluded as its amino acid sequence was incompletely characterized. The PEP-FOLD 3.5 webserver was used to predict the peptide structures from the amino acid sequences of the peptides (Lamiable et al., 2016). This webserver uses a Hidden Markov Model suboptimal sampling algorithm to predict the peptide structures. The resulting peptide structures were used as the starting point for 20 ns molecular dynamics simulations, and the root mean square deviations of the alpha carbon atoms were calculated. The final frames of these molecular dynamics simulations were used for further studies.

### Protein Preparation

The three-dimensional structure of the spike receptor-binding domain (RBD) of SARS-CoV-2 at 2.43 Å resolution (PDB ID: 6M0J) was retrieved from the Protein Data Bank. The protein structure was prepared by removing heteroatoms and water molecules using Discovery Studio (Discovery Studio, 2009). Additionally, energy minimization of the protein structure was performed using the AMBER14 (Case et al., 2014) force field in YASARA software (Krieger et al., 2013). Molecular docking and dynamics studies used this energy minimized protein structure.

### Molecular Docking

The peptides and RBD protein were uploaded as ligand and receptor molecules respectively to the HDOCK, HPEPDOCK, and ClusPro web servers. HDOCK uses a combined template-based and template-free algorithm in an automatic manner (Yan et al., 2017), while HPEPDOCK uses a hierarchical algorithm (Zhou et al., 2018). After docking the peptides to the protein using HDOCK and HPEPDOCK, ClusPro was used to calculate binding energies (Comeau et al., 2004). The top ten peptides with the highest docking scores were selected for further evaluation. These ten peptides were docked to the RBD using the HawkDock web server. For further analysis, the four highest-scoring peptides were chosen based on their binding free energy and docking scores from HawkDock. On the HawkDock server, the HawkRank scoring system, the ATTRACT docking algorithm improved in groups, and MM/GBSA free energy decomposition analysis are implemented on a multipurpose platform (Weng et al., 2019). PyMOL and Discovery Studio (Discovery Studio, 2009) were used for structural analysis of the top four protein-peptide complexes.

### Molecular Dynamics Simulation

Molecular dynamics simulations was performed in YASARA dynamics (Land and Humble, 2018) using the AMBER14 force field (Wang et al., 2004). The docked peptide-protein complexes were initially cleaned, optimized and the hydrogen bond network was oriented. A cubic simulation cell was created with periodic boundary conditions and the TIP3P water model was used (Harrach and Drossel, 2014). The simulation cell was extended by 20 Å in each direction beyond the protein-peptide complexes. The physical conditions of the simulation cells were set at 298 K, pH 7.4, and 0.9% NaCl (Krieger and Vriend, 2015). The initial energy minimization of the simulation cells were conducted by steepest gradient approaches with simulated annealing methods (5,000 cycles). The time step of the simulation was 2.0 fs. Long-range electrostatic interactions were calculated by the Particle Mesh Ewald (PME) method with a cut-off radius of 8.0 Å (Essmann et al., 1995; Krieger et al., 2006; Harvey and De Fabritiis, 2009). The simulation trajectories were saved every 100 ps. The simulations were run for 100 ns at constant pressure and temperature, using a Berendsen thermostat. The simulation trajectories were used to calculate root mean square deviations, root mean square fluctuations, solvent accessible surface areas, radii of gyration, and hydrogen bonds (Adji et al., 2021; Dutta et al., 2021; Obaidullah et al., 2021).

The per residue energy contribution of the peptide-protein structures was calculated using the pyDockEneRes webserver. The first, last, and average structure was extracted from simulations trajectories and utilized as input entry, and the average higher energy from the hotspot residues were tabulated. This tool can be utilized for the identification of the hotspot residues (Romero-Durana et al., 2020).

EresipyDockSCele_vdw=∑sidechainiscEiscele+Eiscvdw=−ΔΔG(Romero-Durana et al.,2020

### Allergenicity and Toxicity Prediction

AllerTOP (Dimitrov et al., 2014a) and AllergenFP (Dimitrov et al., 2014b) webservers were used to predict the allergenicity of the peptides. AllergenFP implements five E-descriptor-based fingerprinting, whilst AllerTOP uses both k-nearest neighbor (kNN) and amino acid E-descriptors to predict the allergenicity of peptides. The ToxinPred (Gupta et al., 2013) webserver was utilized to predict the toxicity of the peptides.

## Results and Discussion

### Molecular Docking

Venom extracted from hymenopteran insects, including the solitary bee *Hylaeus signatus*, is a prominent source of antimicrobial peptides (AMPs). Several α-helical amphipathic AMPs, referred to as HYL, have been identified in the solitary bee, with antimicrobial activity against distinct strains of pathogenic bacteria and fungi as well as the ability to lyse cancer cells (Slaninová et al., 2011; Slaninová et al., 2012; Nešuta et al., 2016). Additionally, HYL had low hemolytic activity, suggesting that it may be safe for use in humans (Nešuta et al., 2016).

Notably, the earlier-discovered antimicrobial peptides, including cecropin-A from *Hyalophora cecropia*, exhibited antiviral activity (Both, 1980). Cecropin-A was shown to have antiviral activity against HSV-1, HIV-1, and JV, whereas melittin from *A. mellifera* had antiviral activity against influenza A viruses, HSV-1, HIV-1, JV, TMV, RSV, VSV, enterovirus, and coxsackievirus (Albiol Matanic and Castilla, 2004). In addition, alloferon 1 and alloferon 2 from *C. vicina* were effective against influenza viruses (Chernysh et al., 2002), TnGlv1 and TnGlv2 from *Trichoplusia ni* were effective against AcMNPV (Moreno-Habel et al., 2012), attC and dptB from *Drosophila melanogaster* were effective against SINV (Huang et al., 2013), and a myristoylated peptide from *H. virescens* was effective against HIV-1 and HSV-1 (Ourth, 2004; Feng et al., 2020). Therefore, we hypothesized that the HYL antimicrobial peptides from the solitary bee could have antiviral activity and consequently devised an *in silico* study to test this hypothesis. We found that several HYL peptides bind the RBD of SARS-CoV-2 *in silico* with favorable binding energy and stable conformations through 100 ns of molecular dynamics simulation.

Molecular docking can be utilized to rapidly assess the binding affinities and modes between a target substrate, such as a protein, and diverse ligands, including small peptides, to assist antiviral drug design (Willett and Glen, 1995). Virtual screening can identify the preferred binding orientation, optimal conformation, and binding sites of protein and peptide molecules (Alonso et al., 2006; Liu et al., 2018). Our computational approach confers improved structural precision and rapid prediction of both the existence and strength of binding through the use of multiple diverse docking algorithms aimed at achieving accuracy (Bartuzi et al., 2017).

**FIGURE 1:**
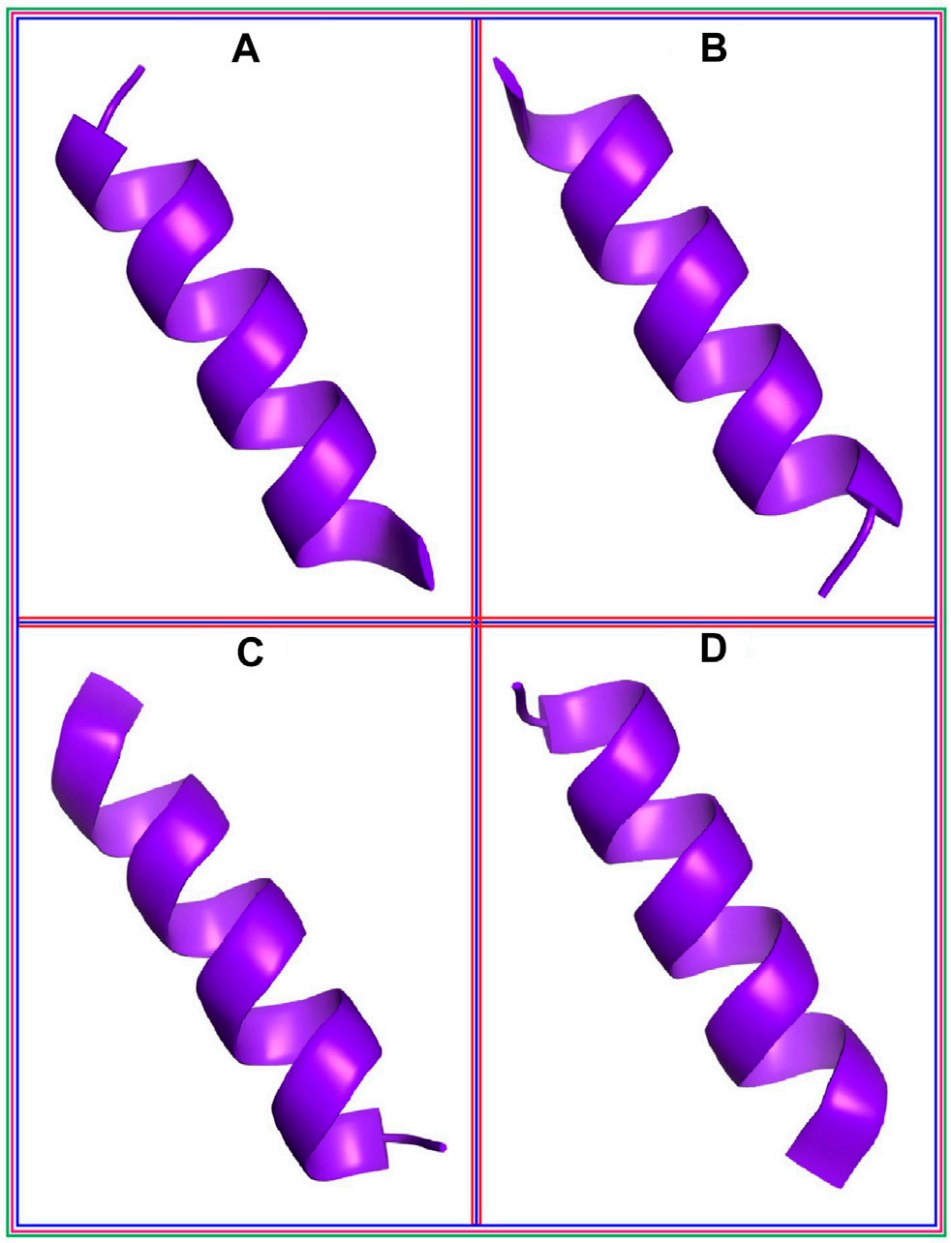
The best four peptide molecules based on the binding free energy in the docking program; **(A)** P2, **(B)** P18, **(C)** P20, and **(D)** P21 peptide molecules.

The predicted peptide structures from PEP-FOLD 3.5 are shown in Supplementary Figure 1. The peptide structures were optimized by molecular dynamics simulations in which the root mean square deviations of the peptides were found to be small (Supplementary Figure 2). After predicting the peptide structures, all 23 peptides were docked to the RBD of SARS-CoV-2 by using the HDOCK, ClusPro, and HPEPDOCK webservers (Supplementary Table 1). Binding energies and/or docking scores were recorded for the ten highest-scoring peptides (Supplementary Table 2). Moreover, the binding free energies and docking scores from HawkDock (Supplementary Table 3**)** were used to select the four highest-scoring peptides (Figure 1). The highest docking score was −2,848.16 kcal/mol for peptide P20, followed by −2,815.13, −2,735.49, and −2,660.34 kcal/mol for P18, P21, and P2, respectively. The maximum binding free energy was −40.38 kcal/mol for the P20 peptide, followed by −37.2, −31.17, and −28.15 for P18, P21, and P2, respectively (Table 1 **)**.

**TABLE 1:** The binding free energy and the dock score of the best four peptide molecules; P2, P18, P20, and P21

Peptide ID	Sequence	Dock score	Binding free energy of complex (kcal/mol)
P2	GIMSSLMKKLKAHIAK	−2,660.34	−28.15
P18	GILSSLWKKLKKIIAK	−2,815.13	−37.2
P20	GILSSLLKKWKKIIAK	−2,848.16	−40.38
P21	GILSSLLKKLKKWIAK	−2,735.49	−31.17

**FIGURE 2:**
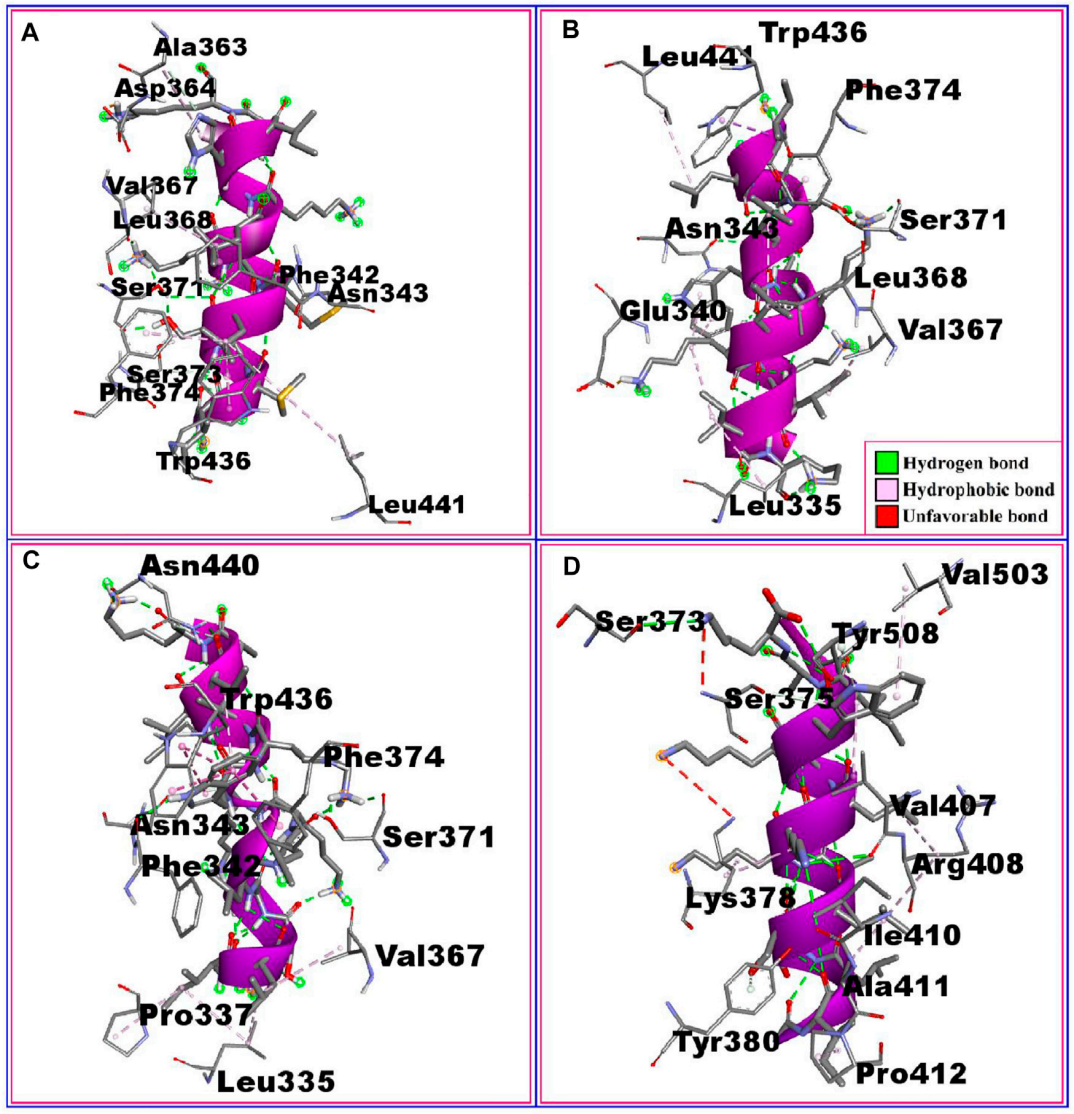
The non-bonded interaction of the P2, P18, P20, P21 peptides and the RBD protein from SARS-CoV-2 at certain simulation times. Here, **(A–D)**, represents the binding interactions between the P2, P18, P20, P21 peptides and the RBD protein after 0ns of simulation time respectively.

The interactions between the RBD protein and the four highest-scoring peptides are shown in Figure 2. The P2:RBD complex had five hydrogen bonds at the RBD residues ALA363, VAL367, SER371, SER373, and ASN343, and six hydrophobic interactions at ASP364, LEU368, PHE374, TRP436, PHE342, and LEU441. The P18:RBD complex formed six hydrophobic interactions at TRP436, PHE374, LEU441, LEU368, VAL367, and LEU335, and three hydrogen bonds at SER371, ASN343, and GLU340. The P20:RBD complex had five hydrophobic interactions at TRP436, PHE374, PRO337, VAL367, and LEU335, and four hydrogen bonds at ASN440, ASN343, PHE342, and SER371. The P21:RBD complex had seven hydrophobic interactions at VAL503, TYR508, LYS378, ARG408, ALA411, TYR380, PRO412 position, one unfavorable bond at SER373 and three hydrogen bonds at SER375, VAL407, ILE410 position **(** Table 2; Figure 2 **)**.

**TABLE 2:** The non-bonded interactions between the P2, P18, P20, P21 peptides and receptor-binding domain (RBD) protein of SARS-CoV-2 after 0ns of simulation times.

Peptide name	Protein	Bond distance (Å)	Interaction category
P2	ALA363	2.68	Hydrogen bond
	ASP364	3.36	Hydrophobic bond
	VAL367	2.77	Hydrogen bond
	LEU368	4.10	Hydrophobic bond
	SER371	2.80	Hydrogen bond
	SER373	2.95	Hydrogen bond
	PHE374	3.31	Hydrophobic bond
	TRP436	4.06	Hydrophobic bond
	PHE342	3.49	Hydrophobic bond
	ASN343	2.73	Hydrogen bond
	LEU441	3.53	Hydrophobic bond
P18	TRP436	3.97	Hydrophobic bond
	PHE374	3.41	Hydrophobic bond
	LEU441	3.39	Hydrophobic bond
	SER371	2.84	Hydrogen bond
	ASN343	3.11	Hydrogen bond
	LEU368	3.79	Hydrophobic bond
	GLU340	2.70	Hydrogen bond
	VAL367	3.77	Hydrophobic bond
	LEU335	3.96	Hydrophobic bond
P20	ASN440	2.94	Hydrogen bond
	TRP436	3.60	Hydrophobic bond
	PHE374	3.44	Hydrophobic bond
	ASN343	2.87	Hydrogen bond
	PHE342	2.77	Hydrogen bond
	SER371	3.69	Hydrogen bond
	PRO337	3.47	Hydrophobic bond
	VAL367	4.02	Hydrophobic bond
	LEU335	3.31	Hydrophobic bond
P21	VAL503	2.72	Hydrophobic bond
	SER373	3.01	Unfavorable bond
	TYR508	3.45	Hydrophobic bond
	SER375	2.49	Hydrogen bond
	VAL407	2.92	Hydrogen bond
	LYS378	3.83	Hydrophobic bond
	ARG408	3.33	Hydrophobic bond
	ILE410	3.11	Hydrogen bond
	ALA411	3.42	Hydrophobic bond
	TYR380	3.56	Hydrophobic bond
	PRO412	3.55	Hydrophobic bond

The interactions between the RBD protein and the remaining six of the top ten peptides are shown in supplementary figure 3**.** The P3:RBD complex formed two hydrogen bonds at ALA344 and ASN343, five hydrophobic interactions at LEU441, LEU335, PHE374, VAL362, and VAL367, and one unfavorable bond at ARG509. The P6:RBD complex had five hydrophobic interactions at LEU441, PHE374, LEU368, VAL367, and LEU335, and six hydrogen bonds at ASN440, ARG509, TRP436, ASN343, PHE342, and GLY339 position. The P16:RBD complex had four hydrophobic interactions at CYS391, CYS525, ALA522, and VAL362, two hydrogen bonds at ASP389 and THR523, one salt bridge at ASP389 and one unfavorable bond at CYS361. The P17:RBD complex had five hydrophobic interactions at LEU335, VAL367, LEU368, PHE342, and TRP436, and three hydrogen bonds at SER371, ASN343, and ASN440. The P22:RBD complex formed five hydrogen bonds at ASN440, ASN343, SER371, GLU340, and ASP364, three hydrophobic interactions at TRP436, VAL367, and LEU335, and one salt bridge at GLU340. The P23:RBD complex formed six hydrophobic interactions at ALA522, THR333, CYS391, CYS525, VAL362, and LEU390 and two hydrogen bonds at GLY526 and ASP389 (Supplementary table 4, Supplementary figure 3).

### Molecular Dynamics

**FIGURE 3:**
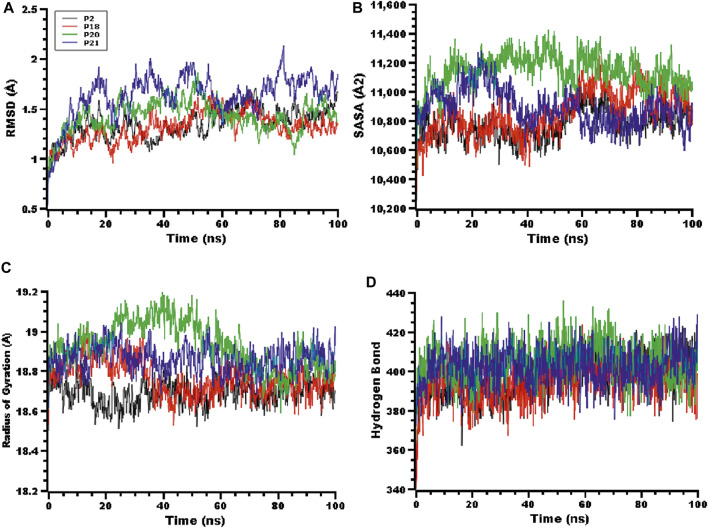
The molecular dynamics simulation of the peptides and RBD complex, here **(A)** root mean square deviation (RMSD) of the alpha carbon atom, **(B)** solvent accessible surface area (SASA), **(C)** radius of gyration (Rg), **(D)** hydrogen bonding of the complexes to estimate their stability in the simulation time.

Molecular dynamics simulations were conducted to explore the structural stability of the docked peptide-protein complexes. The root mean square deviations (RMSD) from the peptide-protein systems were calculated from the simulation trajectories. Figure 3A indicates that the peptide-protein complexes involving the P2, P18, P20, and P21 peptides had an initial upwards RMSD trend, suggesting flexibility of the complexes. The peptide-protein systems subsequently stabilized after 30 ns and maintained their integrity for the remaining 70 ns of the simulations. The P21 complex had a comparatively higher RMSD than the other three peptide complexes, which indicates the more flexible nature of this complex. However, all four peptide-protein complexes had RMSD lower than 2.5 Å, which indicates the stable nature of the complexes over the whole simulations.

The solvent-accessible surface area (SASA) of the complexes was also explored. Figure 3B indicates that the P20 and P21 complexes increased in SASA upon binding with the protein target, and after 30 ns the P21 complex decreased in SASA, maintaining a similar SASA trend to the rest of the complexes. The P20 complex stabilized after 50 ns and maintained its integrity. The radius of gyration (Rg) of the complexes was also explored to understand the compactness of the complexes. High Rg indicates an extended nature whereas a low Rg indicates a more compact structure. Figure 3C indicates that P20 had slightly higher Rg than other complexes which indicates that this complex is less compact. The other complexes had smaller deviations, which indicate the rigid nature of these complexes. Hydrogen bonding can play a crucial role in determining the stability of protein complexes. Figure 3D indicates that all complexes had a stable hydrogen bonding profile over the 100 ns of simulation.

**FIGURE 4:**
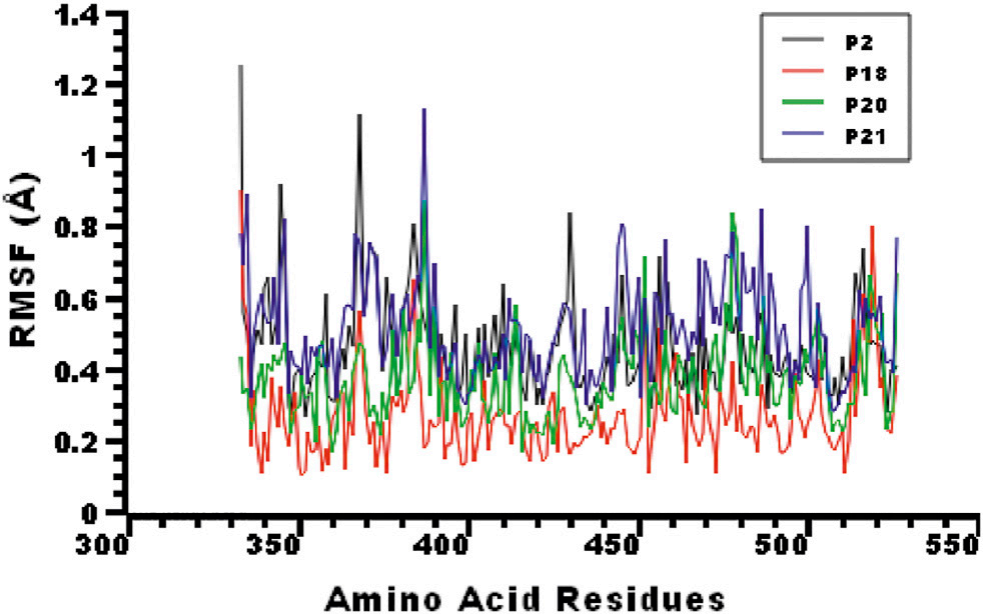
The root mean square fluctuation (RMSF) of the complexes to analyze the flexibility of the amino acid residues.

The root mean square fluctuations (RMSF) of the complexes were explored to understand the flexibility on a residue-by-residue basis. Figure 4 indicates that almost every residue of the complexes had an RMSF lower than 1.4 Å, indicating the stable nature of the protein-peptide systems.

After 100 ns of simulation time, the P2:RBD complex was stabilized by five hydrogen bonds at ASN343, SER371, ASN370, ASP364, and ALA363, as well as five hydrophobic interactions at LEU441, TRP436, LEU368, VAL367, and LEU335. The P18:RBD complex had two hydrogen bonds at SER371 and GLU340, as well as six hydrophobic interactions at TRP436, PHE374, PHE342, LEU368, VAL367 and LEU335. The P20:RBD complex formed two hydrogen bonds at ASN440 and SER371, in addition to three hydrophobic interactions at TRP436, LEU368 and LEU335. The P21:RBD complex had four hydrogen bonds at ILE410, LYS378, TYR508 and PHE374, in addition to three hydrophobic interactions at PRO412, ALA411 and VAL407 (Supplementary table 5, Supplementary figure 4).

In the P2:RBD simulation, the interactions at LEU441, TRP436, ASN343, SER371, LEU368, VAL367, ASP364, and ALA363 were occupied throughout the whole simulation period. Similarly for TRP436, PHE374, SER371, GLU340, LEU368, VAL367, and LEU335 in the P18:RBD simulation, ASN440, TRP436, SER371, and LEU335 in the P20:RBD simulation, and PRO412, ALA411, ILE410, LYS378, VAL407, and TYR508 in the P21:RBD simulation **(** Table 2; Figure 2 **,** Supplementary table 5, Supplementary figure 4).

The active sites of the RBD protein include amino acid residues 340, 374, 375, 378, 403, 420, 477, 478, 499, 543, and 546 (Lan et al., 2020). At the start of the simulation, the P2 peptide bound to the active site of RBD via a hydrophobic interaction with the conserved residue PHE374 (bond distance 3.31360), whereas the P18 peptide interacted with the RBD active site at both PHE374 (Bond distance 3.41730) and GLU340 (bond distance 2.70389) with a hydrophobic interaction and a hydrogen bond respectively. The P20 peptide bound to the active site of RBD at PHE374 (bond distance 3.44294) via a hydrophobic interaction, and the P21 peptide interacted with the RBD active site at SER375 (bond distance 2.49462) and LYS378 (bond distance 3.83247) position with a hydrogen bond and a hydrophobic interaction respectively (Table 2; Figure 2).

After 100 ns of simulation time, the P18 peptide was bound to the active site of RBD at two conserved residues, PHE374 (bond distance 3.44205) and GLU340 (bond distance 2.73681), via a hydrophobic interaction and a hydrogen bond respectively. The P21 peptide interacted with the active site of RBD via LYS378 (bond distance 2.81702) and PHE374 (bond distance 2.87497) via two hydrogen bonds (Supplementary table 5, Supplementary figure 4). These interactions of the peptides with the active site of the RBD indicate that these peptides may inhibit the RBD with high binding affinity.

Previously suggested multifunctional peptides, including ALPEEVIQHTFNLKSQ and DIENLIKSQ from *Bacillus*-fermented soybean, failed to bind to the active site of the RBD. However, our top four peptides bound to the active site. In addition, P18 and P20 had higher binding energy than DIENLIKSQ when employing the same HawkDock server (Padhi et al., 2021). The computationally designed peptide APASMFLGKGDHEILM made no interactions with the active site when docked using the same HPEPDOCK server that we used for our top four peptides (Priya et al., 2021). Another peptide modeling and screening study suggested that AVP0671 can bind to the RBD, albeit not at the active site, and the HDOCK scores were not reported. Meanwhile, our top four suggested peptides not only exhibited active site binding but also had a more favorable HDOCK docking score (Rathod et al., 2020). Antiviral peptides S2P25 and S2P26 were predicted to form one bond at the active site, but had lower docking interaction energies than our P21 and P18 peptides when comparing the Cluspro scores (Chowdhury et al., 2020). Additionally, AVP1795, identified from the computational screening of 645 antiviral peptides, exhibited only one interaction (ARG403) at the active site, which was occupied for ≥90% of the time in MD simulations, whereas our peptides were stably bound over the whole simulation period (Hossain et al., 2021).

Multiple methodology developed to understand the hotspot residues in protein-protein interactions which are based on snapshot from MD simulation sampling (Gohlke et al., 2003). In addition to pyDockEneRes, mm_pbsa.pl tools successfully implemented for hotspot resdiues identifications from cytokines and receptor interface (Du et al., 2020) as well as RBD from SARS-CoV-2 and antibodies (Yang et al., 2021). Moreover, the pyDockEneRes tools enables the hotspot residues identifications from the protein-protein interactions which is key to understand the biological process at molecular level (Romero-Durana et al., 2020). In our study, the P2 peptide and RBD complex, four important residues contribute to overall energy contributions: Leu335, Glu340, Val367, and Phe374; in the P18-RBD complex, hotspot residues include Asp164, Ser373, Ser375, and Trp380 (Table 3). Leu335, Asn343, Asp364, Val367, Ser373, Phe374, and Glu484 were major energy contributing residues in the P20-RBD complex, whereas Leu335, Phe342, Asn343, Asp364, Val367, Ser373, Phe374, and Glu484 were key energy contributing residues in the P22-RBD complex.

**TABLE 3:** The per residue energy contribution from RBD of SARS-CoV-2 where energy contribution was considered <2 kcal/mol.

Complex	Residues	Energy
P2	Leu335	−4.27
	Glu340	−2.28
	Val367	−2.74
	Phe374	−3.37
P18	Asp364	−2.94
	Ser373	−2.21
	Ser375	−2.36
	Trp380	−2.21
P20	Leu335	−3.68
	Asp364	−3.71
	Val367	−3.10
	Trp436	−2.50
P22	Leu335	−5.70
	Phe342	−4.07
	Asn343	−2.60
	Asp364	−4.49
	Val367	−5.96
	Ser373	−2.35
	Phe374	−3.90
	Glu484	−2.21

### Allergenicity and Toxicity Prediction

An allergenic antigen can activate Th2 cells resulting in stimulation of B cells to generate immunoglobulin E (IgE) that binds to FcεRI and activates eosinophils leading to inflammation and tissue shrinkage (Dimitrov et al., 2013). The online AllerTOP tool, which assesses allergens using E-descriptors affined with amino acid attributes, was utilized to predict the allergenicity of our peptides (Dimitrov et al., 2014a). Three distinct web-based approaches were utilized to assess toxicity (Table 4). According to the AllergenFP v1.0 webserver, P2 is a probable allergen, whereas P18, P20, and P21 are probably non-allergenic. The same result was obtained using the AllerTOP v2.0 webserver. In addition, all four peptides (P2, P18, P20, and P21) were categorized as non-toxic by the ToxinPred web server.

**TABLE 4:** The allergenicity and toxicity profiling of the best four-peptide molecules.

Peptide ID	Sequence	Allergenicity prediction		Toxicity prediction through toxinPred
		AllergenFP v.1.0	AllerTOP v. 2.0	
P2	GIMSSLMKKLKAHIAK	Probable allergen	Probable allergen	Non-toxic
P18	GILSSLWKKLKKIIAK	Probable non-allergen	Probable non-allergen	Non-toxic
P20	GILSSLLKKWKKIIAK	Probable non-allergen	Probable non-allergen	Non-toxic
P21	GILSSLLKKLKKWIAK	Probable non-allergen	Probable allergen	Non-toxic

## Conclusion

To develop novel therapeutics against SARS-CoV-2, targeting the spike protein RBD by designing antiviral peptides could identify promising leads. In this study, 23 peptides were docked to the RBD of SARS-CoV-2, leading to the identification of four peptides with high binding affinity. Molecular dynamics studies demonstrated that docked peptides were not ductile in nature, but were instead rigidly bound. Moreover, allergenicity and toxicity profiling of the peptides suggest that they have no allergenic or toxic properties. Finally, our study may facilitate the development of efficient drugs against SARS-CoV-2 through further *in vitro* studies.

## Data Availability

The original contributions presented in the study are included in the article/supplementary material, further inquiries can be directed to the corresponding authors.
